# Metformin induces distinct bioenergetic and metabolic profiles in sensitive versus resistant high grade serous ovarian cancer and normal fallopian tube secretory epithelial cells

**DOI:** 10.18632/oncotarget.23661

**Published:** 2017-12-23

**Authors:** Melissa Hodeib, Martin P. Ogrodzinski, Laurent Vergnes, Karen Reue, Beth Y. Karlan, Sophia Y. Lunt, Paul-Joseph P. Aspuria

**Affiliations:** ^1^ Women’s Cancer Program, Samuel Oschin Comprehensive Cancer Institute, Cedars-Sinai Medical Center, Los Angeles, CA 90048, USA; ^2^ Department of Biochemistry and Molecular Biology, Michigan State University, East Lansing, MI 48824, USA; ^3^ Department of Physiology, Michigan State University, East Lansing, MI 48824, USA; ^4^ Department of Human Genetics, David Geffen School of Medicine, University of California Los Angeles, Los Angeles, CA 90095, USA

**Keywords:** metformin, ovarian cancer, bioenergetics, metabolomics

## Abstract

Metformin is a widely used agent for the treatment of diabetes and infertility, however, it has been found to have anti-cancer effects in a variety of malignancies including high grade serous ovarian cancer (HGSC). Studies describing the mechanisms by which metformin affects HGSC are ongoing, but detailed analysis of its effect on the cellular metabolism of both HGSC cells and their precursor, normal fallopian tube secretory epithelial cells (FTSECs), is lacking. We addressed the effects of metformin and the more potent biguanide, phenformin, on HGSC cell lines and normal immortalized FTSECs. Cell proliferation assays identified that FTSECs and a subset of HGSC cell lines are relatively resistant to the anti-proliferative effects of metformin. Bioenergetic and metabolomic analyses were used to metabolically differentiate the metformin-sensitive and metformin-resistant cell lines. Bioenergetically, biguanides elicited a significant decrease in mitochondrial respiration in all HGSC cells and FTSECs. However, biguanides had a greater effect on mitochondrial respiration in metformin sensitive cells. Metabolomic analysis revealed that metformin and phenformin generally induce similar changes in metabolic profiles. Biguanide treatment led to a significant increase in NADH in FTSECs and HGSC cells. Interestingly, biguanide treatment induced changes in the levels of mitochondrial shuttle metabolites, glycerol-3-phopshate (G3P) and aspartate, specifically in HGSC cell lines and not in FTSECs. Greater alterations in G3P or aspartate levels were also found in metformin sensitive cells relative to metformin resistant cells. These data identify bioenergetic and HGSC-specific metabolic effects that correlate with metformin sensitivity and novel metabolic avenues for possible therapeutic intervention.

## INTRODUCTION

Ovarian cancer remains the leading cause of gynecologic cancer-related death in women despite widespread efforts to improve surgical procedures and therapeutic targets [1]. In 2015, 21,290 new ovarian cancer diagnoses were made in the United States, and >66% (14,180) of these women died of the disease [2]. High grade serous carcinoma (HGSC) accounts for over half of ovarian cancers and carries the worst overall prognosis [1]. Standard treatment for ovarian cancer involves surgical debulking with the goal of no gross residual disease, followed by combination platinum and taxane chemotherapy. Despite advances there have been only modest improvements in the overall 5- and 10-year relative survival rates which remain 46% and 35%, respectively [1]. Repurposing low-toxicity drugs may help improve the progression free and overall survival rates [1]. Also, understanding the mechanism of how low toxicity drugs affect cancer cells may reveal additional therapeutic targets.

Metformin, a biguanide drug with a low toxicity profile, has been widely used to treat diabetes and fertility [3, 4]. In 2005, Evans et al reported a reduced incidence of cancer in diabetic patients receiving metformin, which led to recognition of the drug in cancer-related research [4]. Another large prospective study found that diabetic women treated with metformin have a lower risk of dying of most invasive cancers compared to non-metformin users [5]. Metformin and phenformin, two biguanide drugs traditionally used to treat diabetes, have now been associated with improved survival rates in many different cancer types including non-small cell lung, breast and ovarian cancers [6–8]. Due to safety concerns, phenformin has been removed from the pharmaceutical market for use in humans [9]. However, recent studies have shown that phenformin treatment may have some utility in treating cancer with a shorter treatment schedule that reduces the risk of severe side effects [6].

As anti-diabetic medications, biguanides primarily act as an insulin sensitizers, decrease blood glucose levels, and reduce gluconeogenesis in the liver [10]. Both increased levels of insulin and glucose have been associated with tumor growth and poor overall prognosis in different cancer types [10]. Therefore, the ability of biguanides to lower both glucose and insulin levels may indirectly contribute to its anti-tumor effects. In addition to these indirect effects, biguanides are also posited to directly affect cancer cell proliferation via inhibition of Complex I within the electron transport chain [11]. Indeed, it was recently found that metformin accumulates in tumors and induce metabolic changes similar to that seen *in vitro* [12]. The bioenergetic stress induced by metformin inhibits proliferation and was largely thought to be mTOR dependent [13, 14]. However, metformin inhibition of mTOR has been shown to vary between different studies and cell types, with no correlation to its anti-proliferative effects [12, 15].

Preclinical studies focusing on the effect of metformin on HGSC have identified its anti-proliferative effects [8, 12, 16]. These data and epidemiological evidence have led to clinical trials assessing the use of metformin in both neoadjuvant and post-surgical settings for HGSC [12, 17]. However, a molecular characterization of cell lines widely used to study HGSC revealed that they are, in fact, not likely to represent the disease [18]. Also, growing evidence has pointed to the fallopian tube secretory epithelial cells (FTSEC) as the origin of HGSC [19]. FTSECs have not been metabolically characterized, and their response to biguanides are unknown. Extensive metabolic characterization of HGSC cells has also not been reported. Therefore, to assess the metabolic and potential anti-proliferative effect of biguanides in HGSC, we performed bioenergetic and metabolomic analysis on a panel of clinically relevant HGSC lines and normal cell of origin controls. We find that a subset of HGSC cell lines as well as normal FTSECs are relatively resistant to the anti-proliferative effects of metformin. Also, these effects do not correlate with the ability of metformin to inhibit AMPK/mTOR signaling. Bioenergetic analysis revealed that metformin sensitivity largely correlated with a greater inhibition of oxygen consumption rate. Also, metabolomic analysis identified specific alterations in HGSC cells versus normal FTSECs that also correlate with metformin sensitivity.

## RESULTS

### Biguanides inhibit HGSC cell proliferation

**Figure 1:**
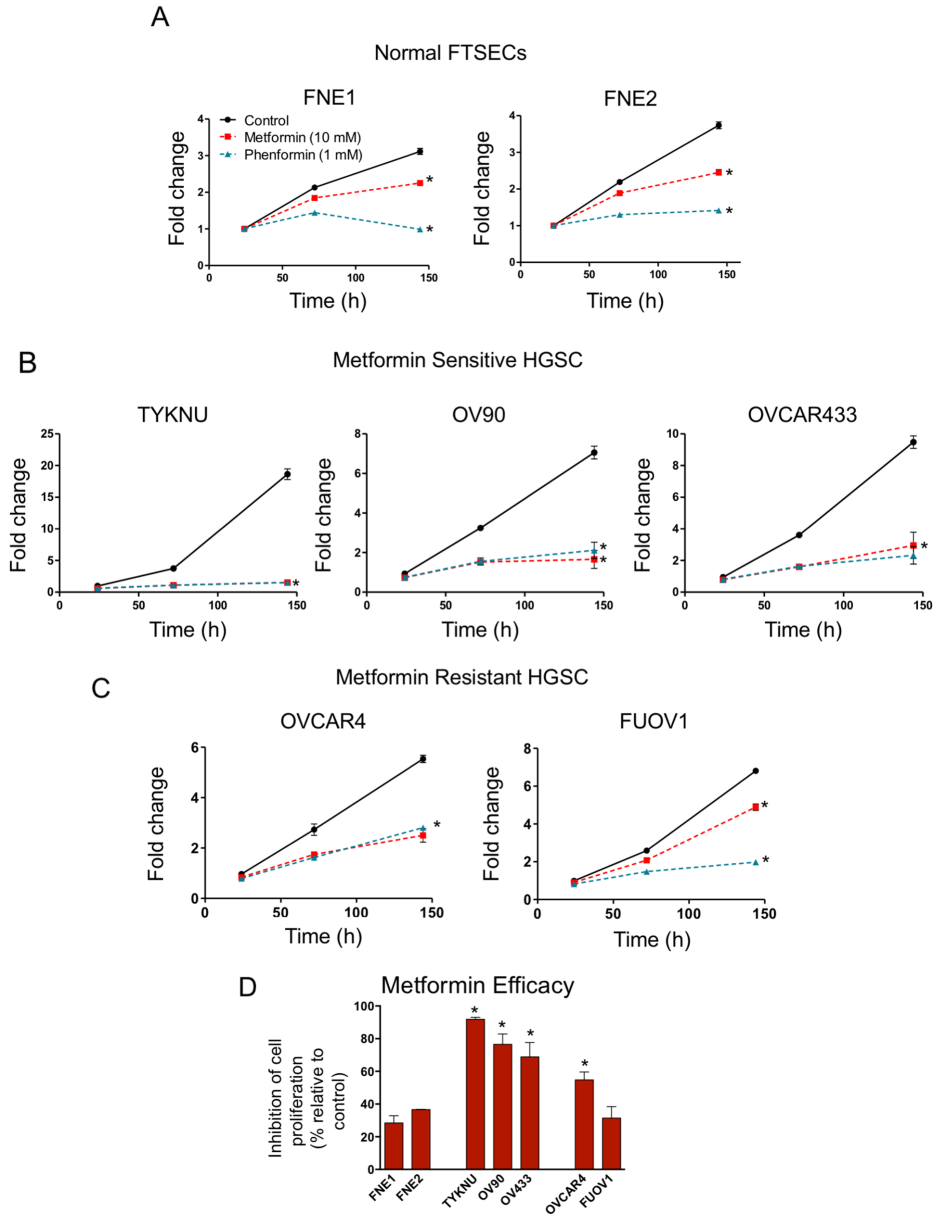
The effects of biguanides on 2-D cell proliferation of HGSC and normal FTSEC cell lines **(A)** Normal FTSECs, **(B)** metformin sensitive and **(C)** metformin resistant cells grown in 2-D were treated with the indicated doses of metformin, phenformin, or vehicle control at 24 h for 5 days. Cell proliferation was assessed at indicated time points by Celltiter Glo. Proliferation is displayed relative to vehicle control at 24h. **(D)** Metformin efficacy calculated based on metformin treatment relative to control after 5 days of treatment. ^*^denotes significant inhibition relative to control treatment (p-value <.01).

**Figure 2:**
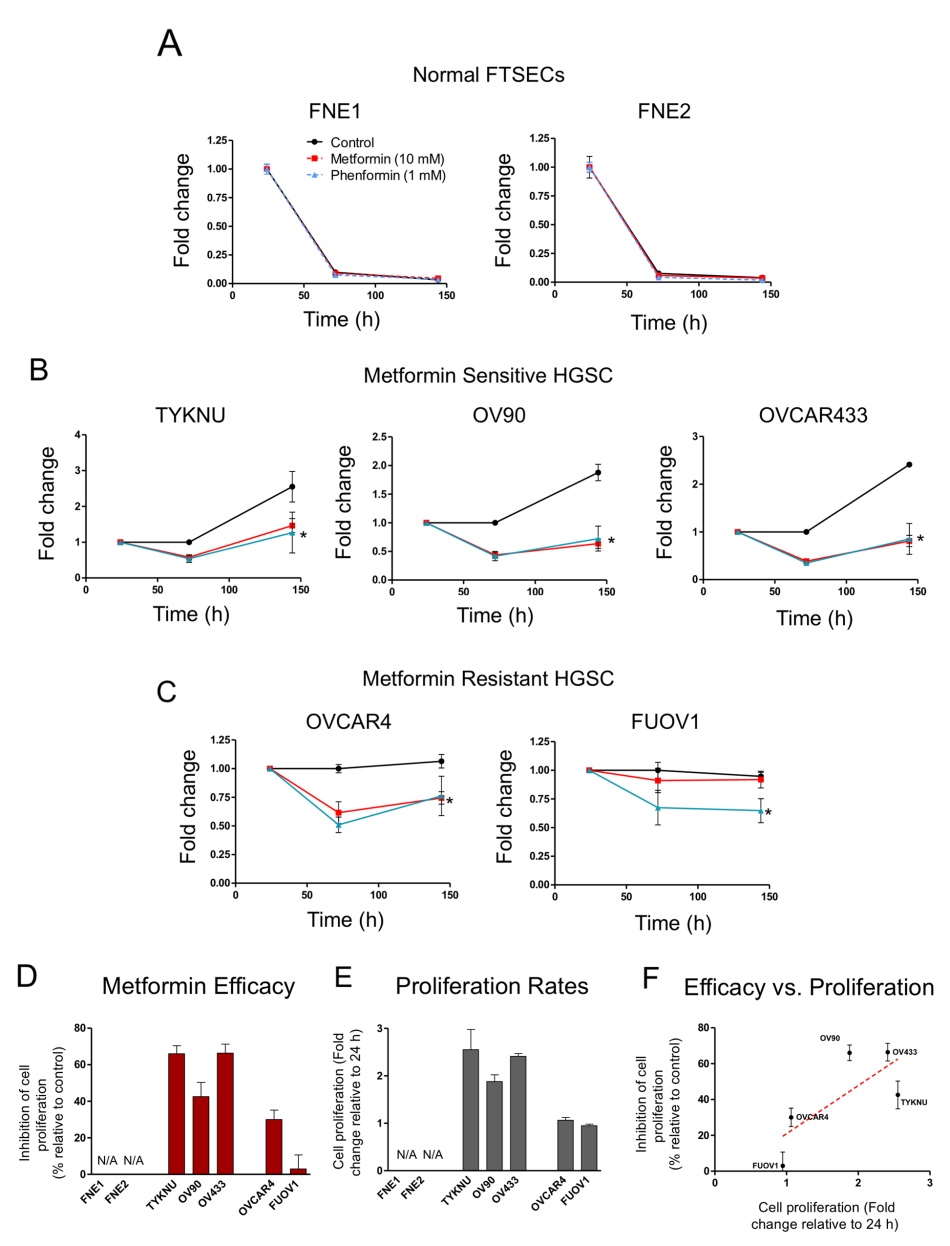
The effects of biguanides on 3-D cell proliferation of HGSC and normal FTSEC cell lines **(A)** Normal FTSECs, **(B)** metformin sensitive and **(C)** relatively metformin resistant cells grown in ultra-low attachment 3D conditions. Cell proliferation was assessed at indicated time points by Celltiter Glo 3D. Proliferation is displayed relative to vehicle control at 24h. **(D)** Metformin efficacy calculated based on metformin treatment divided by control treatment at 5 days of treatment. **(E)** Proliferation at 6 days relative to 24 h. **(F)** Plot of metformin efficacy versus cell proliferation rates. Dotted red line is best-fit trend line of all data points and statistically significant to be non-zero (p-value <0.01). ^*^denotes significant inhibition relative to control treatment (p-value <0.01).

We examined the effect of metformin and phenformin on normal FTSEC and HGSC proliferation in 2-D growth conditions. We analyzed a panel of HGSC cell lines (FUOV1, OV90, OVCAR4, OVCAR433, and TYKNU), which were previously characterized as suitable HGSC models given their genetic makeup (i.e. *TP53* mutation, copy-number profile, and low frequency of non-synonymous mutations in protein-coding genes) [19]. Normal TERT-immortalized fallopian tube non-ciliated epithelium cell lines, FNE1 and FNE2, were used as normal controls [20]. Normal FTSECs and HGSCs were treated with either metformin, phenformin, or vehicle control (Figure 1). In FTSECs, metformin treatment led to a modest growth inhibition (∼30-40%), while phenformin completely inhibited cell proliferation (Figure 1A & 1D). In HGSCs, phenformin also significantly inhibited cell proliferation (Figures 1B & 1C). However, metformin treatment of HGSC cell lines revealed two subgroups; Metformin-sensitive (TYKNU, OV90, and OVCAR433) and metformin-resistant (OVCAR4 and FUOV1) (Figure 2B–2D). Metformin completely inhibited the cell proliferation of metformin-sensitive cells (Figure 1B & 1D), while metformin-resistant cells responded similarly to normal FTSECs, with OVCAR4 being slightly more sensitive (Figure 1C & 1D).

We also assessed the effect of metformin and phenformin on anchorage independent 3D growth. Cells were grown in ultra-low attachment plates for 24 hours to form cellular aggregates and then treated with metformin, phenformin, or vehicle. As expected, FNE1 and FNE2 were unable to survive anchorage-independent conditions (Figure 2A). However, all HGSC cell lines formed stable cell aggregates and continued to survive after 6 days (Figure 2B & 2C). Cells were less proliferative in 3D conditions compared to 2D conditions (Figures 1 & 2). Generally, cell lines that were highly proliferative in 2D (TYKNU, OV90, and OV433) were also more proliferative than other cells in 3D (Figure 1 & 2). The effects of metformin in 3D were similar to those observed in 2D; the growth of TYKNU, OV90, and OVCAR433 was significantly inhibited by metformin, whereby OVCAR4 and FUOV1 were not (Figure 2B–2D). We noticed that the 3D growth of metformin resistant cells was significantly slower than that of the metformin sensitive cells (Figure 2E). Indeed, there was a statistically significant (p-value =.0037) inverse relationship between metformin resistance and 3D cell proliferation rate (Figure 2F). These data indicate that normal FTSECs and a subset of HGSC cell lines are relatively metformin resistant.

### The effect of metformin on proliferation does not correlate with phospho-S6K levels

**Figure 3:**
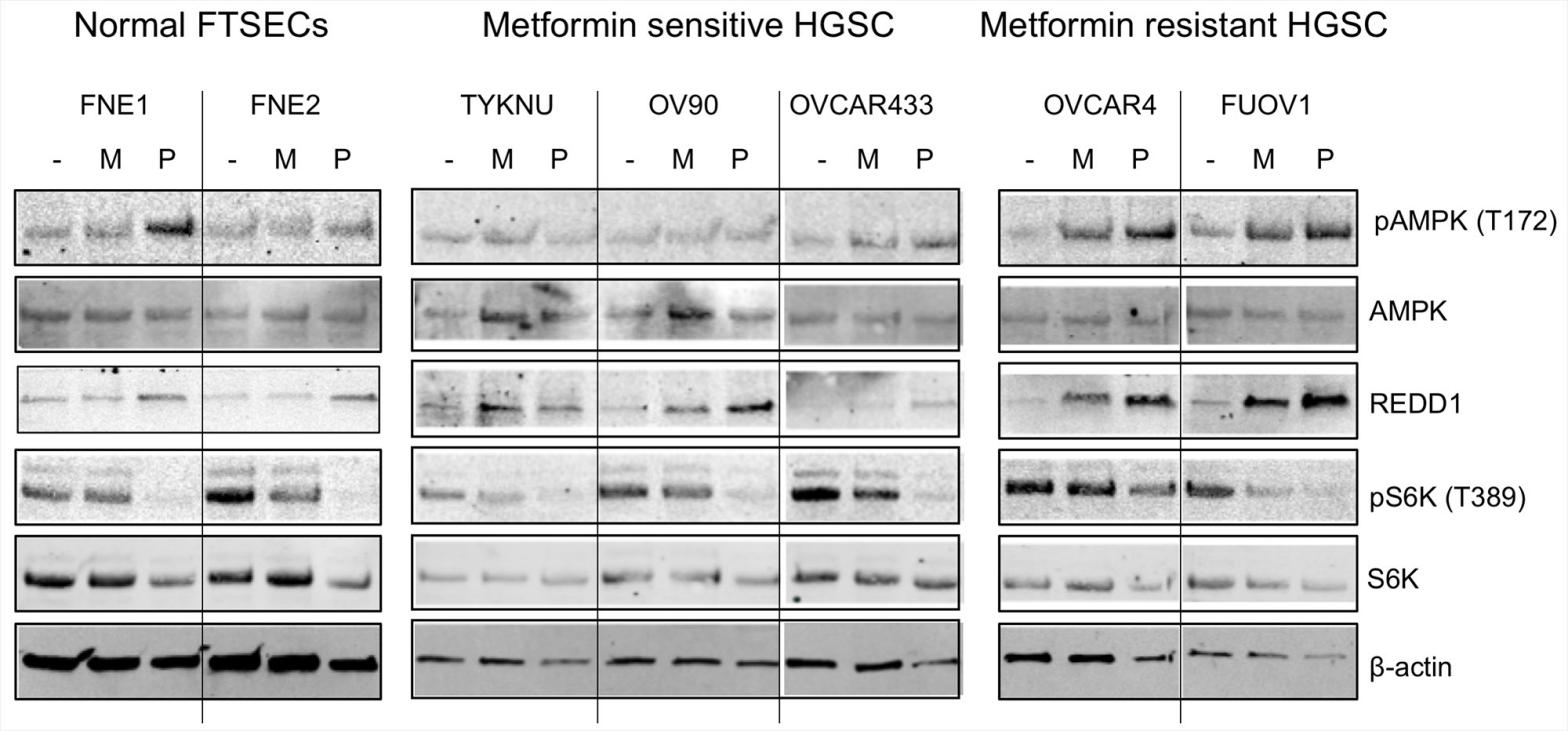
The effects of biguanides on mTOR signaling in HGSC and normal FTSEC cell lines Cell lines were treated with metformin (10 mM), phenformin (1 mM), or vehicle control for 24 hours. Western blot analysis of phospho-AMPK (T172), AMPK, phospho-S6K (T389), S6K, REDD1, and β-actin as a loading control.

The effect of biguanide treatment on proliferation in other cell types has been primarily described through inhibition of mTOR activation via the upregulation of AMPK activity or REDD1, both well-established mTOR inhibitors [6, 13, 14]. To identify possible differences between metformin-resistant and metformin-sensitive cells, we examined the effects of biguanides on the mTOR signaling pathway in FTSEC and HGSC cells. Both biguanides induced AMPK Thr172 phosphorylation (pAMPK) only in the metformin-resistant lines (OVCAR4 and FUOV1) (Figure 3). Phenformin, but not metformin, also induced pAMPK in FNE1 (Figure 3). We further performed time course experiments addressing the effect of metformin and a potent inducer of AMPK, AICAR, in the metformin-sensitive, OV90, and metformin-resistant, FUOV1, cell lines. Metformin was able to induce a subtle increase in pAMPK in OV90 after 6 hours which decreased significantly by 48 h (Supplementary Figure 1A). This is juxtaposed to the dramatic and sustained increase of pAMPK in FUOV1 cells (Supplementary Figure 1A). AICAR was able to induce phospho-AMPK levels in both cell lines, however to a much lesser extent in OV90 (Supplementary Figure 1A). Metformin sensitivity also did not correlate with the expression of the upstream modulator of AMPK activity, LKB1, nor the expression of the metformin transporter, OCT1 (Supplementary Figure 1B&1C). Western blot analysis of REDD1 found that both biguanides induced REDD1 protein levels in all HGSC cells, while only phenformin treatment led to elevated levels of REDD1 in normal FTSEC cells (Figure 3). We determined whether REDD1 was also transcriptionally upregulated by performing qRT-PCR analysis. Indeed, both biguanides induced similar levels of REDD1 mRNA in HGSC cells but not in normal FTSECs (Supplementary Figure 2). We then assessed mTOR activity via the phosphorylation status of the mTOR downstream target, S6 kinase (S6K), by western blot. Phenformin significantly decreased phospho-S6 kinase (pS6K) levels in all cell lines, indicating mTOR inhibition (Figure 3). In contrast, metformin decreased pS6K levels in only two cell lines, TYKNU (metformin-sensitive) and FUOV1 (metformin-resistant) (Figure 3). Therefore, these data suggest that phenformin is a more potent inhibitor of mTOR activity than metformin, even at doses where metformin has anti-proliferative effects. Together, these data are in line with other studies that suggest upregulation of pAMPK, REDD1, and inhibition of mTOR activity does not correlate with metformin sensitivity in cancer cell lines [12, 15]. It also indicates that the anti-proliferative effects of metformin may be at least partially attributable to mechanisms other than mTOR inhibition.

### HGSC cell lines have altered bioenergetic profiles compared to normal FTSECs

**Figure 4:**
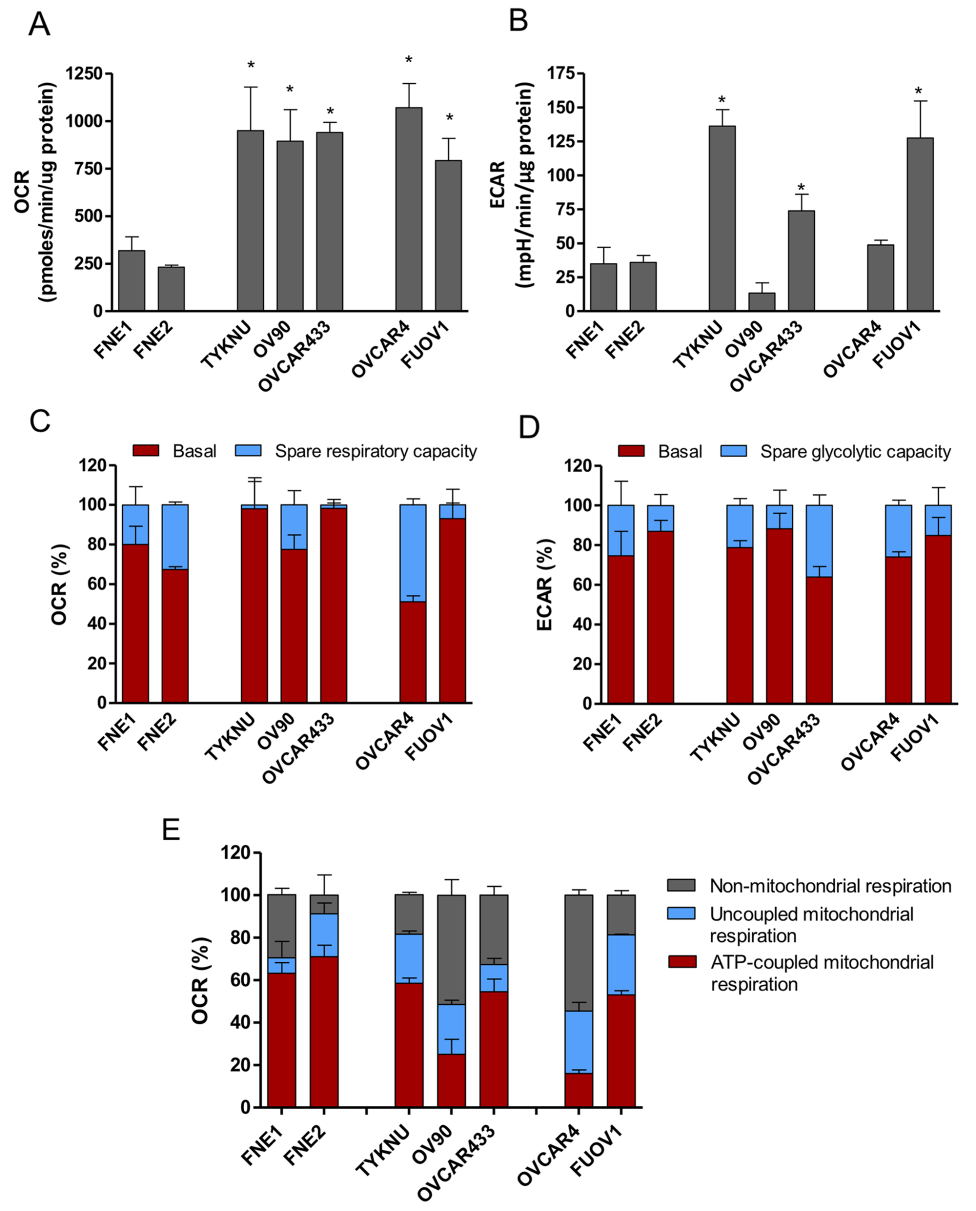
Bioenergetic analysis of HGSC and normal FTSEC cell lines **(A-E)** Oxygen Consumption Rate (OCR) (A, C, E) and Extracellular Acidification Rate (ECAR) (B & D) measurements were obtained using an extracellular flux analyzer (Seahorse Bioscience). Basal OCR (A) and ECAR (B) rates were obtained prior to addition of Oligomycin A to derive Spare Glycolytic Capacity (D) and ATP-coupled OCR (E), and FCCP to uncouple mitochondria for maximal OCR. (C) Spare respiratory capacity was calculated by taking the difference between the maximal OCR and basal OCR. Percentages are relative to maximal respiration. (D) Spare Glycolytic Capacity was derived by taking the difference between maximal ECAR and basal ECAR. Percentages are relative to maximal ECAR. (E) Non-mitochondrial respiration was calculated as the residual OCR after treatment with Rotenone/myxothiazol that inhibits Complex I. Uncoupled mitochondrial respiration was calculated as the difference between OCR following Oligomycin A treatment and OCR following Rotenone/myxothiazol treatment. All three values are shown as percentages relative to baseline OCR.

Since the effects of metformin could not be fully explained by mTOR inhibition, we sought to characterize the metabolic and bioenergetic effects of biguanide treatment. We initially profiled the baseline bioenergetic activities of FTSECs and HGSC cell lines utilizing the Seahorse bioanalyzer to assess oxygen consumption rate (OCR), a key indicator of mitochondrial activity and cellular respiration, as well as the extracellular acidification rate (ECAR), an indicator of glycolysis. Analysis of the baseline OCR revealed that HGSC cell lines display a significantly increased OCR relative to normal cells (Figure 4A). In addition, most HGSC cell lines, except OVCAR4 and OV90, have an increased baseline ECAR relative to normal cells (Figure 4B). These data imply that HGSC cells have elevated cellular respiration and increased glycolysis as compared to their cell of origin counterparts.

Since HGSC cells displayed significantly elevated OCR and ECAR, we determined whether these cells were functioning at their maximal respiratory and glycolytic capacities. FCCP, a mitochondrial uncoupler, induces maximal respiration by transporting protons across the mitochondrial membrane leading to depolarization of the membrane potential and rapid consumption of O_2_. This maximal OCR is used in conjunction with basal OCR to calculate spare respiratory capacity. FNE1 and FNE2 mitochondria were functioning at 80% and 67% capacity with 20% and 37% spare respiratory capacity, respectively (Figure 4C). While a few HGSC cell lines (FUOV1, OVCAR433, and TYKNU) were functioning at near maximal capacity (>90% capacity, <10% spare respiratory capacity), other HGSC cell lines (OV90, OVCAR4) were functioning at significantly lower (<70% capacity, >30% spare respiratory capacity), or similar capacities relative to normal (Figure 4C). Therefore, no general trend in spare respiratory capacity could be identified between metformin-resistant cells, metformin-sensitive cells, and normal controls. To calculate maximal glycolytic capacity, oligomycin, an ATP synthase inhibitor, was used to induce a bioenergetic shift towards glycolysis (maximal ECAR). Similar to spare respiratory capacity, there were no significant differences between HGSC and control cells in spare glycolytic capacities (Figure 4D).

We further assessed other facets of mitochondrial function including the percentage of respiration devoted to ATP production (ATP-coupled), proton leak (ATP-uncoupled), and non-mitochondrial respiration. OCR measurements during sequential treatment of cell lines with oligomycin (ATP synthase inhibitor) and rotenone/myxothiazol (Complex I and III inhibitors, respectively) allow for these parameters to be defined. Normal cell lines, FNE1 and FNE2, have greater than 60% of their total respiration dedicated to ATP synthesis (Figure 4E). However, all HGSC cell lines tested demonstrate significantly less ATP-coupled OCR than controls with the majority of their respiration being allocated towards uncoupled and non-mitochondrial respiration (Figure 4E). This phenomenon of elevated non-ATP-coupled respiration in cancer versus normal cells has also been observed in breast cancer [21]. Altogether these data suggest that HGSC cells are more bioenergetic, while contributing a smaller fraction of their total respiration towards ATP-synthesis compared to normal cells.

### Biguanides significantly inhibit oxygen consumption while increasing glycolysis in both normal FTSECs and HGSC cells that can be exploited in low glucose conditions

**Figure 5:**
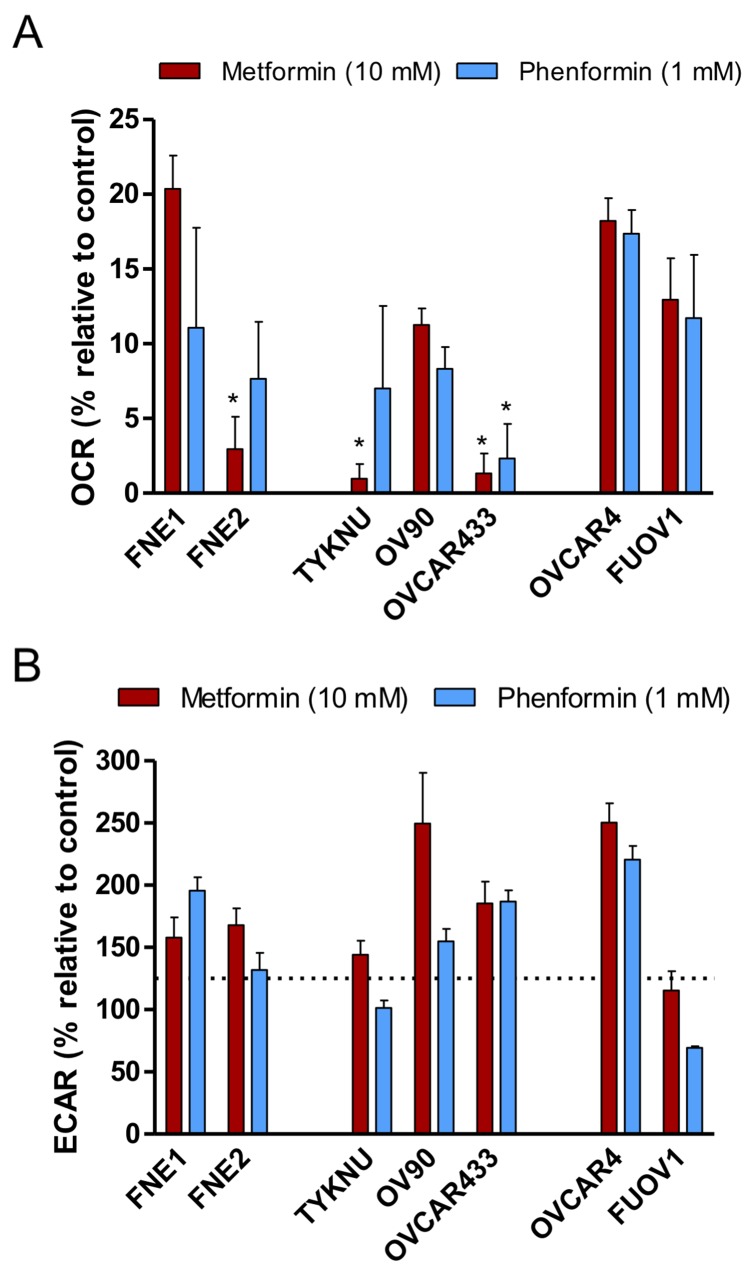
The effects of biguanides on the bioenergetics profiles of HGSC and normal FTSEC cell lines **(A and B)** Cells were treated with Metformin (10 mM), Phenformin (1 mM), or control vehicle for 24 hours prior to analysis by the extracellular flux analyzer. (A) Basal OCR relative to control treated cells. ^*^ denotes p-value < 0.05 as determined by Tukey’s multiple comparison test. (B) Basal ECAR relative to control treated cells. Dotted line indicates the level of a statistically significant change in ECAR (p-value < 0.01).

To assess the effects of metformin and phenformin on mitochondrial function, cell lines were incubated for 24 hours with either metformin, phenformin, or vehicle control prior to Seahorse bioanalysis. Treatment with metformin or phenformin significantly decreased respiration (>70% of control OCR) in both HGSC and normal cells at similar levels. However, the metformin-resistant cells (OVCAR4 and FUOV1), still had a significantly higher OCR (>10%) than the metformin-sensitive cells (<10%) (Figure 5A). This implies that oxidative phosphorylation is less inhibited in metformin-resistant cells as compared to metformin-sensitive cells. Therefore, metformin and phenformin decrease overall oxygen consumption and utilization for ATP-synthesis. Biguanides are also more potent in affecting these processes in metformin-sensitive cells versus metformin-resistant cells.

Previous studies have shown that metformin inhibition of oxygen consumption leads to a subsequent compensatory increase in aerobic glycolysis to compensate for the energy deficit in some cell lines [22, 23]. Therefore, we examined the effect of metformin and phenformin on the ECAR of HGSC and normal cells. Both FNE1 and FNE2 had significant ECAR increases upon treatment of metformin or phenformin relative to control (Figure 5B). Most HGSC cell lines also had elevated ECAR upon metformin treatment except FUOV1 (Figure 5B). Similarly, phenformin treatment increased ECAR in most HGSC cell lines except TYKNU and FUOV1 (Figure 5B). These data confirm previous reports that metformin and phenformin generally inhibit oxygen consumption, but the induction of aerobic glycolysis is governed by other factors [22, 23]. Also, under these conditions, these bioenergetic effects do not discriminate between metformin-sensitive and metformin-resistant cells.

**Figure 6:**
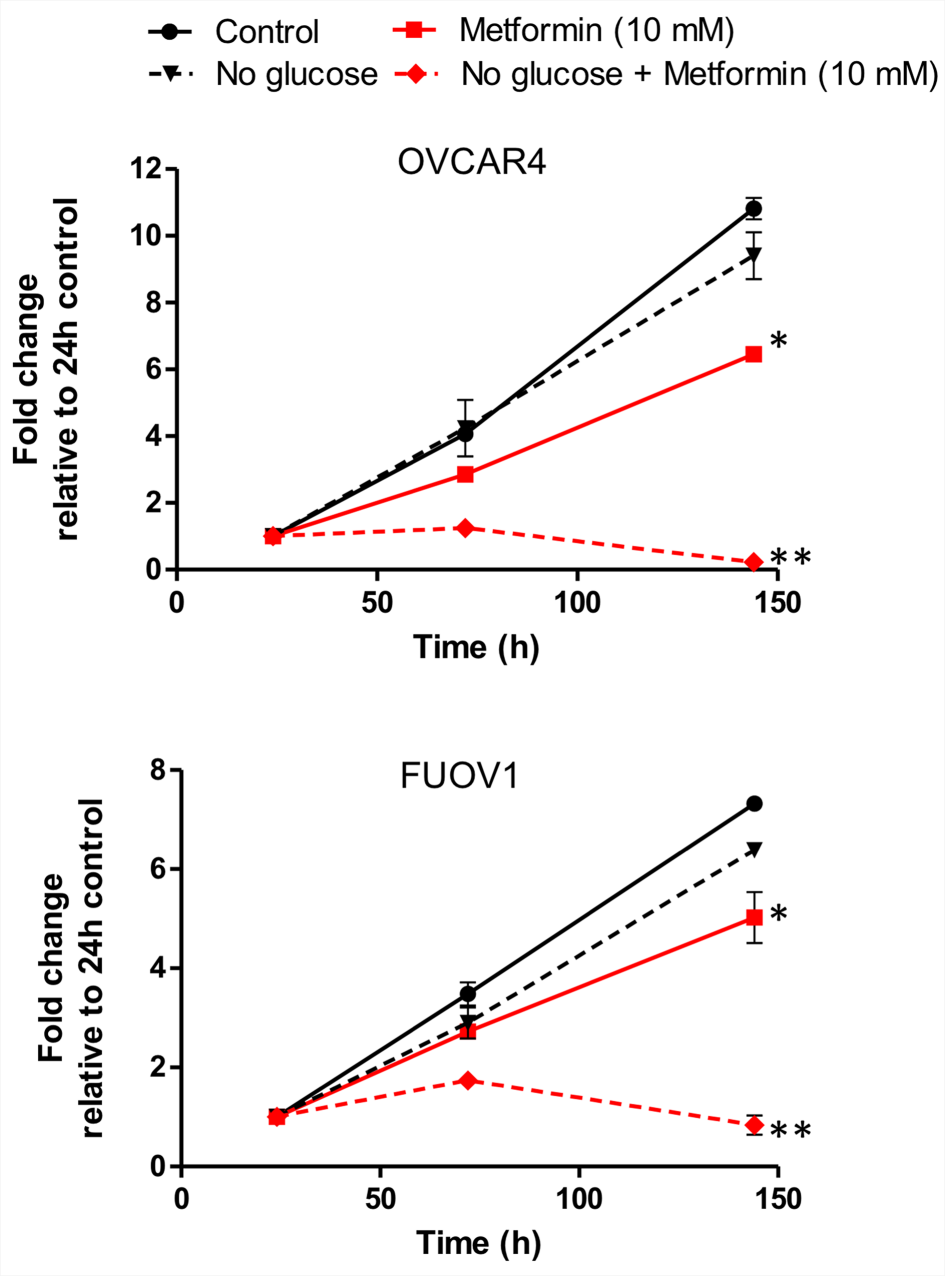
Glucose deprivation sensitizes metformin resistant cell lines FUOV1 and OVCAR4 to metformin treatment Cells were grown in standard glucose or limited glucose conditions were treated with vehicle or metformin (10 mM). Cell proliferation was assessed at 24 h, 72h, and 144 h. Proliferation is depicted relative to 24 h for each treatment. ^*^ denotes p-value < 0.01 relative to control. ^**^ denotes p-value <0.01 relative to metformin in standard glucose media.

A previous study in ovarian cancer indicated that metformin resistance can be overcome by reducing glucose concentration, thereby demonstrating the inhibitory effect of hyperglycemia on the actions of metformin [16]. To address whether glucose served as a protective molecule in metformin resistant cells, we cultured FUOV1 and OVCAR4 cells in media with standard (10 mM) or low (0.1 mM) glucose concentrations and treated cells with metformin, phenformin, or control. There was no significant difference in cell proliferation between untreated glucose and low glucose media after 6 days (Figure 6A & 6B). However, metformin and phenformin treatment significantly inhibited proliferation in both cell lines under low glucose conditions compared to standard media (Figure 6A & 6B). These data further support the previous study that adequate levels of glucose are required for biguanide resistant cells to survive.

### Metabolomic analysis of metformin and phenformin treatment reveals cancer cell specific metabolite fluctuations

**Figure 7:**
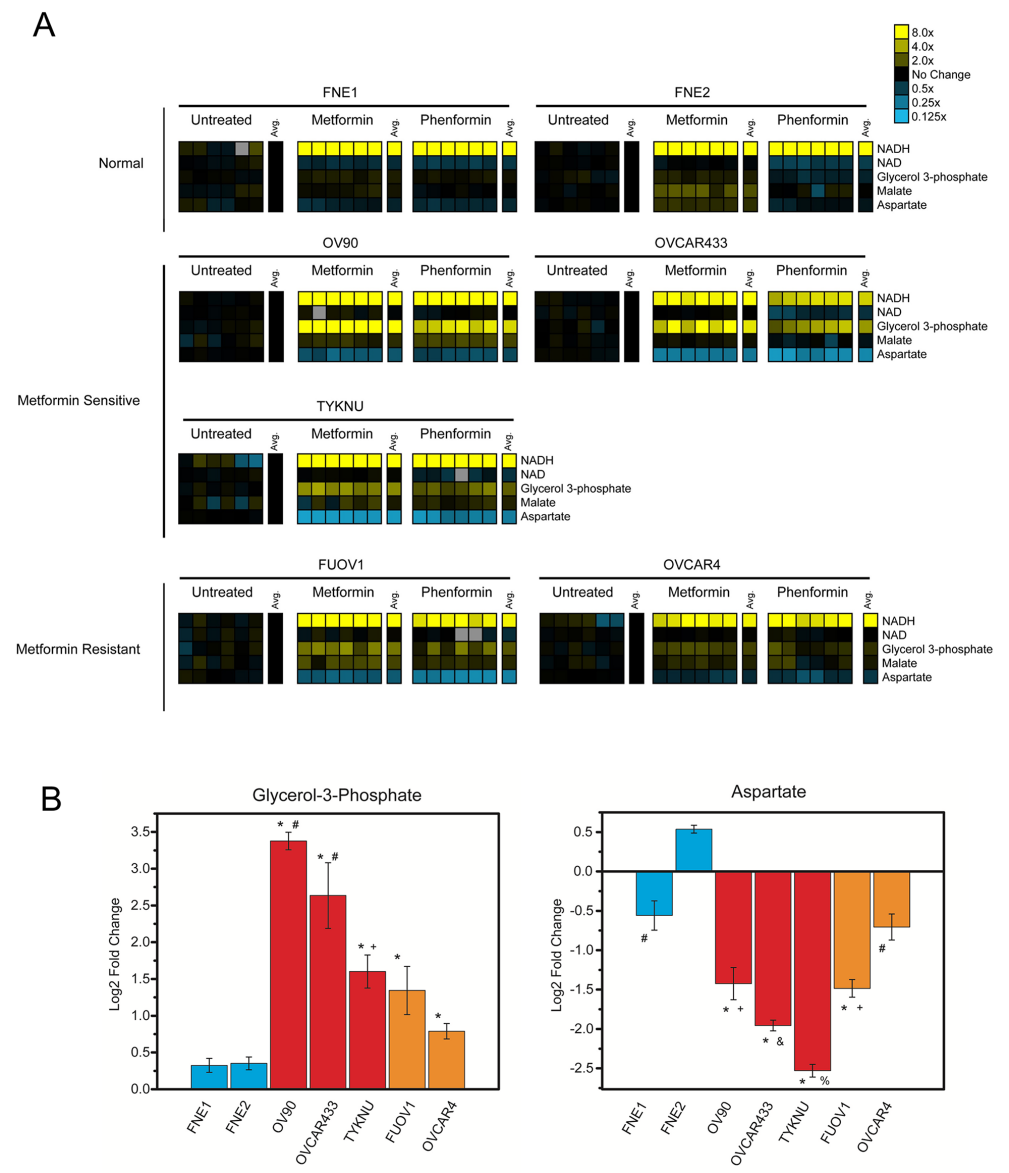
The cancer-specific effects of biguanides on mitochondrial shuttle metabolites **(A)** Metabolite analysis. Cells were treated with metformin (10 mM), phenformin (1 mM), or vehicle control for 24 hours and subjected to targeted mass spectrometry analysis. Metformin and phenformin treatments shown relative to untreated control. Yellow and blue heatmaps indicate increased or decreased levels relative to control, respectively. Data normalized by cell number. Complete metabolite changes located in Supplementary Figure 2. **(B)** Quantification of G3P and aspartate fold changes induced by metformin treatment. Values listed as log2 fold change in metabolite abundance for metformin treated versus control normal FTSECs (blue), metformin-sensitive (red), and metformin-resistant (orange) cells. For G3P: ^*^p-value < 0.05 vs normal cell lines by Games-Howell test, ^#^p-value < 0.05 vs TYKNU, FUOV1, and OVCAR4 by Games-Howell test, ^+^p-value < 0.05 vs OVCAR4 by Games-Howell test. For aspartate: ^*^p-value < 0.05 vs normal cell liens by Games-Howell test, ^#^p-value < 0.05 vs FNE2 by Games-Howell test, ^+^p-value < 0.05 vs OVCAR4 by Games-Howell test, ^&^p-value < 0.05 vs OV90, FUOV1, and OVCAR4 by Games-Howell test, and ^%^p-value < 0.05 vs OV90, OVCAR433, FUOV1, and OVCAR4 by Games-Howell test.

**Figure 8:**
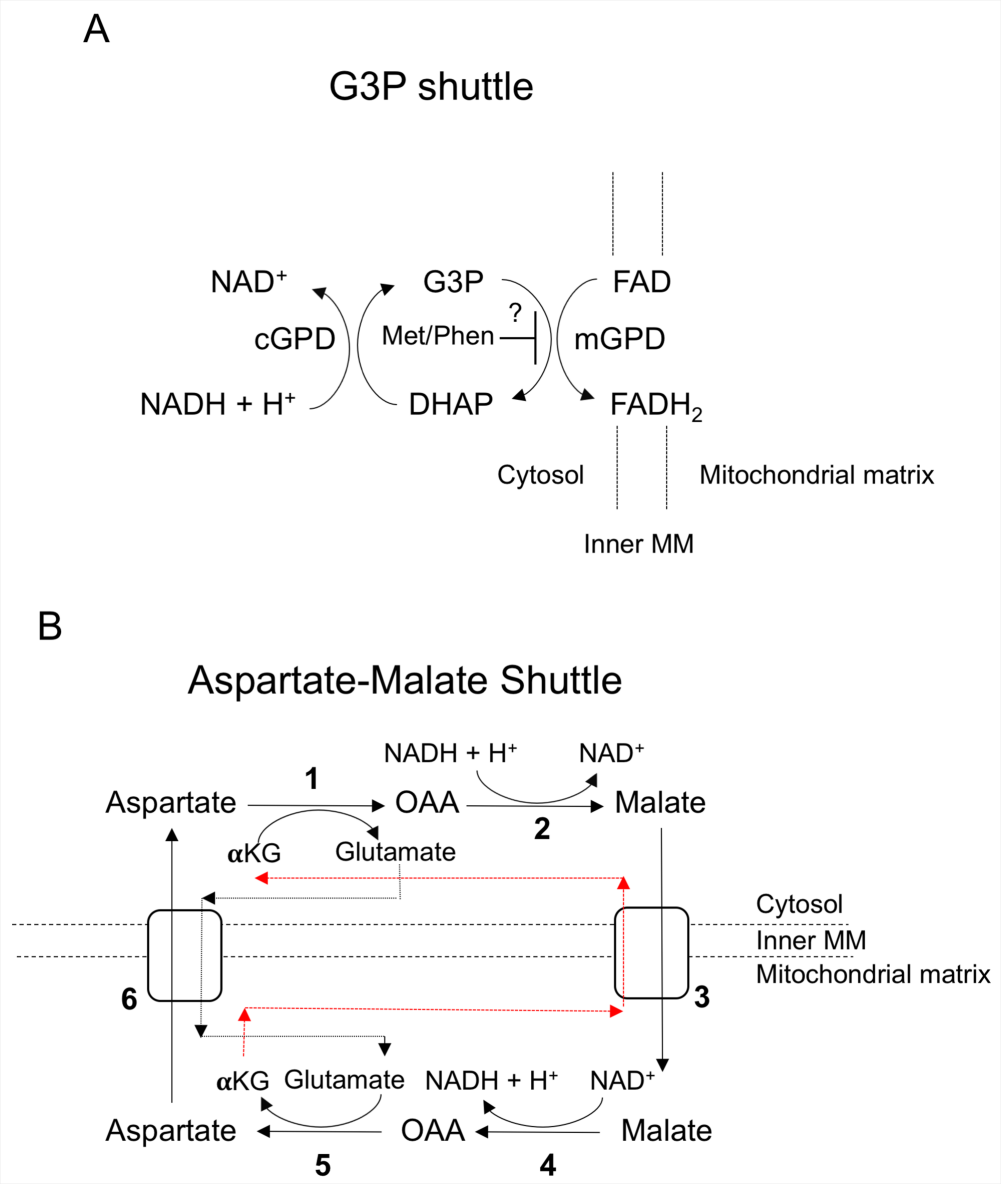
Mitochondrial shuttles **(A)** The glycerol-3-phosphate shuttle. cGPD = cytosolic glycerol-3-phosphate dehydrogenase, mGPD – mitochondrial glycerol-3-phosphate dehydrogenase. **(B)** The malate-aspartate shuttle. Numbers indicate the following enzymes and transporters: (1) Cytosolic aspartate aminotransferase (2) Malate dehydrogenase 1 (3) Malate-alpha-ketoglutarate antiporter (4) Malate dehydrogenase 2 (5) Mitochondrial aspartate aminotransferase (6) Glutamate-aspartate antiporter. Black and red dashed lines indicate the flow of α-ketoglutarate and glutamate between the cytosol and mitochondrial space.

Due to the significant effect of biguanides on mitochondrial function, we were interested in examining the effect of biguanides on central carbon metabolism. We performed metabolomic analysis on both normal FTSECs and all HGSC cell lines (Figure 7, Supplementary Figure 3, Supplementary Table 1) Similar to a previous study, metformin and phenformin generally induce similar metabolic changes in all cell lines tested (Figure 7A, Supplementary Figure 3, Supplementary Table 1) [7]. Treatment with either molecule induced significantly elevated levels of NADH relative to controls in both HGSC and normal FTSEC cells, consistent with Complex I inhibition by biguanides (Figure 7A, Supplementary Figure 3, Supplementary Table 1). In general, biguanide treatment of most cell lines including FNE2 cells also resulted in the depletion of tricarboxylic acid (TCA) cycle intermediates citrate and α-ketoglutarate (Supplementary Figure 3 and Supplementary Table 1). Treatment with biguanides also caused variable depletion of nucleotide triphosphates between cell lines depending on the specific treatment. Treatment with phenformin caused relative depletion of adenosine triphosphate (ATP), cytidine triphosphate (CTP), and uridine triphosphate (UTP) in OVCAR433 and TYKNU, but not of other NTPs in FUOV1 or OV90. Treatment with metformin caused depletion of CTP specifically in FNE2 and FUOV1, as well as depletion of UTP in OV90, OVCAR44, and TYKNU. Neither treatment caused a significant depletion of guanosine triphosphate (GTP) in any of the cell lines studied (Supplementary Figure 3 and Supplementary Table 1). The only metabolite specifically and significantly altered in metformin-sensitive cells versus metformin-resistant cells was the nucleoside deoxyuridine (Supplementary Figure 4). Interestingly, we identified biguanide-induced alterations that were particular to all HGSC cells tested and not normal FTSECs. Specifically, in HGSC cells, metformin and phenformin treatment caused a significant elevation in glycerol-3-phosphate (G3P) and a decrease in aspartate levels relative to controls (Figure 7A&7B). Interestingly, the metformin-sensitive cell lines generally displayed greater effects on either G3P accumulation or aspartate depletion than the metformin-resistant cell lines, especially OVCAR433 (Figure 7A&7B). G3P is primarily involved in the glycerol-phosphate shuttle, which in addition to the malate-aspartate shuttle, allows the movement of electrons from cytosolic NADH to the mitochondria for entry into the electron transport chain (Figure 8A&8B) [24]. The metabolite data suggests that the glycerol-phosphate and malate-aspartate shuttle are perturbed by biguanides thereby leading to an accumulation of G3P and depletion of aspartate. Given that this effect does not occur in the normal FTSECs and is more pronounced in metformin-sensitive cells, it appears that biguanide treatment may specifically affect these mitochondrial shuttles in HGSC cells.

### Aspartate and pyruvate supplementation rescue the anti-proliferative effects of metformin on cell growth

**Figure 9:**
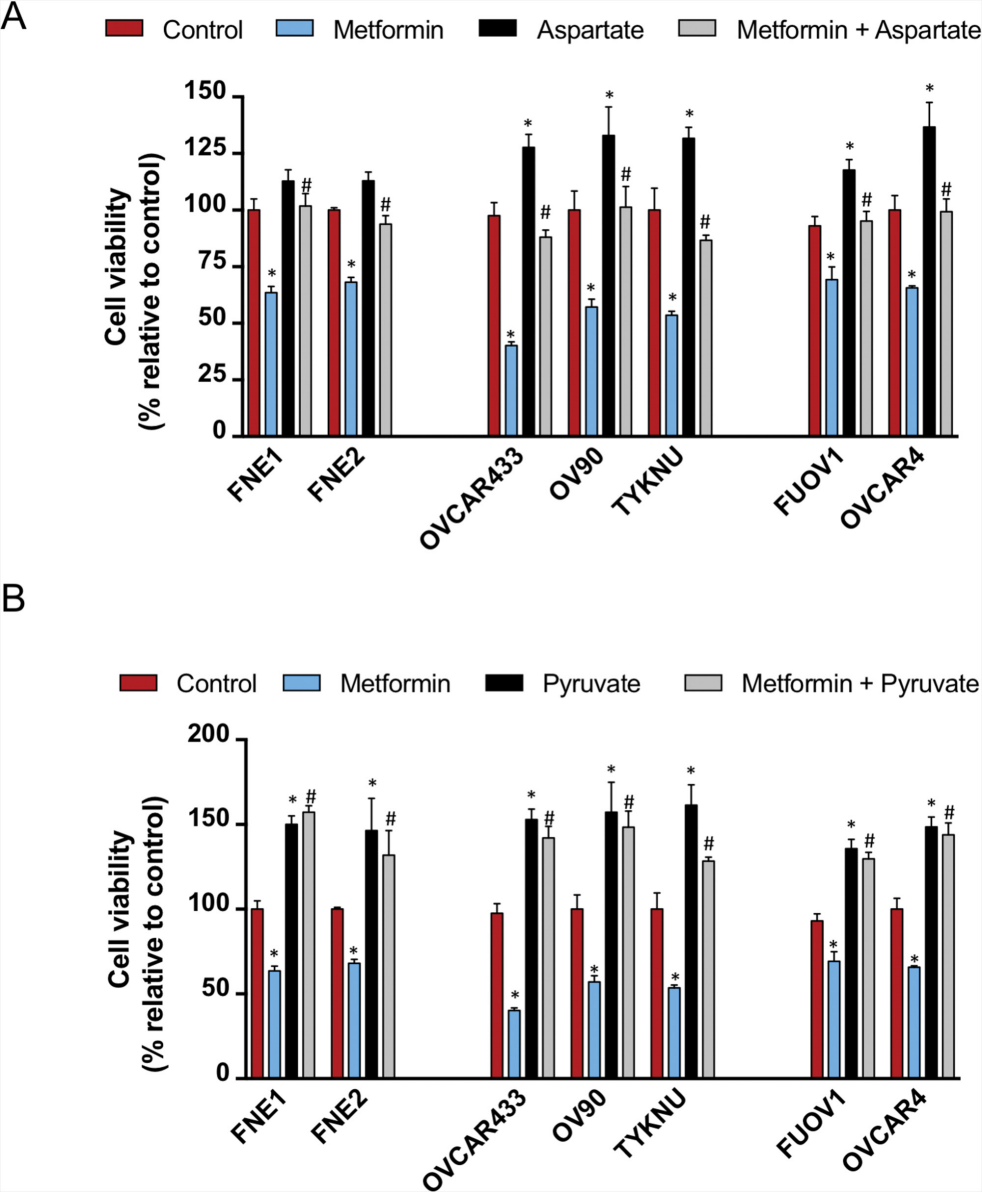
Asparate and pyruvate supplementation inhibits the effects of metformin on cell proliferation Cells were seeded and after 24 h were treated with either control, metformin (10 mM) with or without **(A)** aspartate (100 uM) or **(B)** pyruvate (1 mM). Cell proliferation was assessed after an additional 72 h by Celltiter Glo. Proliferation is displayed relative to vehicle control. ^*^ denotes p-value < 0.01 relative to control. ^#^ denotes p-value < 0.01 relative to metformin treatment.

Given that metformin treatment results in a significant decrease in aspartate levels, we tested whether supplementation of cells with aspartate would rescue the anti-proliferative effects. We treated cells simultaneously with either control, aspartate (100 uM), metformin (10 mM), or both aspartate and metformin for 72 h and assessed cell proliferation (Figure 9A). Treatment with aspartate alone had significant effects on the growth of all HGSC cell lines tested (Figure 9A). Aspartate had a minimal and non-significant effect on the growth of normal FTSEC cell lines (Figure 9A). Combinatorial treatment of all cell lines tested with aspartate and metformin resulted in a diminished effect of metformin, bringing cell viability close to control levels (Figure 9A). Therefore, aspartate supplementation diminishes the metformin effect as previously reported [15].

Previous studies have also shown that providing an alternative carbon source such as pyruvate can overcome the effects of metformin in cancer cell lines [15]. We also treated cells with either control, pyruvate (1 mM), metformin (10 mM), or both pyruvate and metformin for 72 h and assessed cell proliferation. Strikingly, pyruvate treatment had a significantly positive effect on the growth of all normal FTSEC and HGSC cell lines (Figure 9B). Also, pyruvate significantly diminished the anti-proliferative effect of metformin on all cell lines, greater than the effect of aspartate (Figure 9B). This is in line with the report from Gui et al that pyruvate is a more powerful suppressant of metformin’s ability to inhibit cell growth than aspartate.

## DISCUSSION

Multiple studies in different cancers have demonstrated that metformin and phenformin have a wide-ranging impact on cancer metabolism [7, 21, 22]. In a Src-inducible model of breast cancer, both biguanides were found to deplete TCA cycle intermediates as expected from Complex I inhibition [7]. Similar results were found using isolated mitochondria from breast cancer cell lines illustrating that biguanides indeed directly affect mitochondrial function [21]. Biguanide inhibition of TCA cycle activity was also found in NSCLC and colon cancer cell lines [22]. Interestingly, analysis of the effects of biguanides on breast cancer stem cells yielded a different metabolic profile; levels of all ribonucleotide and nucleotide triphosphates (NTPs) were significantly decreased, but no effects were seen on the TCA cycle [7]. In line with these results, we found that biguanides elicited similar effects on metabolites in the TCA cycle and/or NTPs in the HGSC cell lines tested (Supplementary Figure 3). In addition, this is the first study that characterizes the bioenergetics and metabolism of immortalized FTSECs, the purported cell of origin of HGSC [19]. Given that previous biguanide studies on ovarian cancer have not included FTSECs, we were able to identify metabolic effects induced by biguanides that were only seen in transformed HGSC cells. The most significant of these effects was the perturbation of metabolites involved with mitochondrial shuttles, the G3P and malate-aspartate shuttle (Figure 7A). Since NADH is unable to cross the mitochondrial membrane, shuttles exist to transfer electrons from cytosolic NADH to the electron transport chain (ETC) via the reduction of other molecules that can cross into the mitochondria. Mechanistically, the G3P shuttle functions in the following manner: Cytosolic G3P dehydrogenase 1 (cGPD) converts dihydroxyacetone phosphate (DHAP) to G3P by oxidizing NADH to NAD^+^. G3P is then converted back to DHAP by mitochondrial G3P dehydrogenase 2 (mGPD) to produce FADH_2_ that donates its electrons to the ETC (Figure 8A). The malate-aspartate shuttle utilizes malate and aspartate as electron carriers that are shuttled between the cytosol and mitochondria via exchange transporters (Figure 8B). These systems appear to be perturbed by biguanides in transformed HGSC cells as evidenced by elevated levels of G3P and depletion of aspartate. Interestingly, mGPD was found to be a direct target of biguanide inhibition in rats [25]. Whether biguanides inhibit the G3P and malate-aspartate shuttles directly in human cells has not been determined.

Recent evidence has shown that metformin accumulates within ovarian tumors and induces aspartate depletion [12]. This and other studies have posited that metformin prevents the mitochondria from adaptive nutrient utilization since metformin treatment can be rescued by providing alternative fuel sources such as pyruvate or increased amounts of glucose [12, 15, 16]. We have also confirmed the protective effect supplementation of glucose, aspartate, and pyruvate has against metformin (Figure 6 and 9). One caveat of our study is that cells were grown in RPMI media containing supraphysiologic levels of glucose (10 mM vs ∼1-5mM) and higher levels of metformin (10 mM) than cells *in vivo* would be exposed to. However, the metabolomic changes upon metformin treatment seen in our *in vitro* data overlaps significantly with the changes seen in other *in vivo* studies, thereby suggesting translatability of our results [12, 15, 16]. Since biguanide treatment of most HGSC cell lines resulted in depleted nucleotides, increases in glycolysis (as shown by lactate and ECAR), and significant inhibition of ATP-linked OCR, it follows that there is an increased need for glucose to provide the carbons required to replenish nucleotides and ATP via the pentose-phosphate shunt and glycolysis, respectively. Therefore, the ability of metformin to lower blood glucose levels and directly inhibit adaptive nutrient utilization in cancer cells imply a multi-faceted mechanism explaining the efficacy of this anti-tumor agent.

In summary, our study characterizes the metabolic and anti-proliferative effects of biguanides on HGSC cells and its cell of origin, FTSECs. Biguanides significantly inhibit the ETC and accumulate NADH in all cell lines implying that biguanides are also able to enter normal FTSEC cells and act on its direct target, Complex I. However, the anti-proliferative effects of metformin, but not phenformin, are HGSC cell specific and do not correlate with inhibition of mTOR activity. Metabolomic analysis revealed HGSC specific alterations in the levels of mitochondrial shuttle metabolites, aspartate and G3P, thereby illustrating that these processes are of particular importance, and possibly overactive in cancer cells. Alterations in these metabolites also correlate well with the anti-proliferative efficacy of metformin. The activity of these shuttles in HGSC cells versus normal FTSECs have not been described and are worth investigating. Further detailed analysis of the metabolic pathways perturbed in biguanide sensitive cells (i.e. deoxyuridine metabolism) as well as resistance mechanisms in metformin resistant cells may reveal additional metabolic therapeutic targets. Additionally, since this study identifies that metformin induces deleterious effects specifically in HGSC cells not seen in normal FTSECs and its low toxicity profile, its use as a preventative measure for HGSC should be taken into consideration.

## MATERIALS AND METHODS

### Cell lines and reagents

FUOV1, OVCAR4, OV90, OVCAR433, and TYKNU were obtained as previously described [26]. FNE1 and FNE2 (TERT-immortalized normal FTSECs) were a kind gift from Dr. Tan Ince (University of Miami) [20]. Metformin, phenformin, and ultra-low attachment plates were obtained from Sigma-Aldrich. HGSC cells were grown in RPMI 1640 + 10% FBS + 1% penicillin/streptomycin. FNE1 and FNE2 were grown in FOMI media [20] then switched to RPMI 1640 + 10% FBS + 1% penicllin/streptomycin 72 hours prior to assays.

### Mitochondrial function and glycolysis

2x10^4^ cells were plated into 24 well XF plates (Seahorse bioscience). Oxygen consumption rate (OCR) and extracellular acidification rate (ECAR) were measured using an XF24 Extracellular Flux Analyzer (Seahorse Bioscience) in unbuffered DMEM assay medium supplemented with 1 mM pyruvate, 2 mM glutamine and 11 mM glucose. OCR and ECAR were measured before and after the sequential addition of 0.5 μM oligomycin, 0.5 μM FCCP and 1 μM of rotenone/myxothiazol. Values were normalized to protein concentration using a Bradford assay (Bio-Rad). Mitochondrial respiration was calculated as the difference between total and rotenone/myxothiazol rates. Maximal respiration was the response to FCCP. ATP-linked respiration was the oligomycin-sensitive respiration while uncoupled respiration was the difference between oligomycin and rotenone/myxothiazol rates.

### Cell proliferation assay

1 × 10^3^ cells/well were seeded in triplicate on a 96-well plate and treated with metformin (1 mM or 10 mM), phenformin (100μM or 1 mM), aspartate (100 uM), pyruvate (1 mM) or vehicle control (RPMI). To assess cellular viability, cells were subjected to the CelltiterGlo assay (Promega). Luminescence was read on a GloMax luminometer.

### Spheroid formation assay

1 × 10^3^ cells/well were seeded in triplicate in an ultra-low attachment 96-well plate and incubated overnight. Next day cells were treated with indicated doses of metformin, phenformin, or control for 72 hours. Viability was assessed by CelltiterGlo 3D assay.

### Western blot analysis

Western blot was performed as previously described [27]. Briefly, cell lysates were collected in RIPA buffer supplemented with protease inhibitor cocktail and phosSTOP (Roche) and phosphatase inhibitor cocktail (Roche). 30 μg of pre-cleared cell lysate and 4x laemmli buffer were boiled for 10 minutes. Boiled lysates were run on a 4-20% gradient gel (BioRad) and transferred to a PVDF membrane. After blocking in 5% milk/TBS-T, blots were incubated overnight with primary antibody towards AMPK (Cell Signaling), phospho-AMPK (Cell Signaling), REDD1 (Protein Tech), S6K (Cell Signaling), phospho-S6K (Cell Signaling), LKB1 (Santa Cruz) and β-actin (Sigma Aldrich). Blots were washed with TBS-T and incubated with secondary antibodies. Blots were scanned using the LiCOR Odyssey system.

### qRT-PCR analysis

RNA extraction was performed using the RNeasy Mini Kit (Qiagen). RNA was reverse transcribed to cDNA using the Quantitect Reverse Trancription Kit (Qiagen). For qRT-PCR, 50 ng of cDNA was mixed with primers towards REDD1 (Forward 5’-ACAGTTCTAGATGGAAGACC-3’, Reverse 5’-ACAGTTCTAGATGGAAGACC-3’ or RPL32 (Forward 5’-GTGCAACAAATCTTAC-TGTG, Reverse 5’- CTGCCTACTCATTTTCTTCAC).

### Metabolite extraction and analysis

Cells were cultured in 6-well plates with or without metformin (10 μM) or phenformin (1 μM) treatment for 24 hours, and extracted at 80% confluency. Medium was aspirated, and each well was washed with 2ml saline (pH 7.5). Saline was aspirated, and cells were quenched with 500 μl of -75°C HPLC-grade methanol in each well. After adding 200 μl of HPLC-grade water to each well, cells were scraped with a cell lifter. All contents of each well was collected in a 1.7-ml microcentrifuge tube. Chloroform (500 μl at −20°C) was added to each tube and vortexed for 10 min at 4°C. Extracts were centrifuged at 17,000 × g for 15 min at 4°C. The upper aqueous phase containing polar metabolites was collected in a separate microcentrifuge tube and evaporated under a stream of nitrogen. Metabolites were resuspended in 100 μl of HPLC-grade water immediately before analysis by mass spectrometry. The metabolites were analyzed using a Waters Xevo TQ-S mass spectrometer coupled to an H-Class UPLC system. Metabolites were separated by polarity using a Supelco Ascentis Express C18 column (2.7 μm particle size, 5 cm × 2.1 mm). LC parameters are as follows: autosampler temperature, 5 °C; injection volume, 5 μl; column temperature, 50 °C; flow rate over 11 min: *t* = 0, 0.4 ml min^−1^; *t* = 2, 0.3 ml min^−1^; *t* = 3, 0.25 ml min^−1^; *t* = 5, 0.15 ml min^−1^; *t* = 9, 0.4 ml min^−1^; *t* = 11, 0.4 ml min^−1^. The LC solvents were solvent A: 10 mM tributylamine and 15 mM acetic acid in 97:3 water:methanol (pH 4.95); and solvent B: methanol. Elution from the column was performed over 11 min with the following gradient: *t* = 0, 0% B; *t* = 1, 0% B; *t* = 2, 20% B; *t* = 3, 20% B; *t* = 5, 55% B; *t* = 8, 95% B; *t* = 8.5, 95% B, *t* = 9, 0% B; *t* = 11, 0% B. Mass spectra were acquired using negative-mode electrospray ionization operating in multiple reaction monitoring (MRM) mode. The capillary voltage was 3,000 V, and cone voltage was 50 V. Nitrogen was used as cone gas and desolvation gas, with flow rates of 150 l h^−1^ and 600 l h^−1^, respectively. The source temperature was 150 °C, and desolvation temperature was 500 °C. Argon was used as collision gas at a manifold pressure of 4.3 × 10^-3^ mbar. Collision energies and source cone potentials were optimized for each transition using Waters QuanOptimize software. Data analysis was performed using MAVEN [28, 29]. Metabolite measurements were normalized by cell counts.

## SUPPLEMENTARY MATERIALS FIGURES AND TABLE





## Abbreviations

ATP: adenosine triphosphate
cGPD: cytosolic glycerol-3-phosphate dehydrogenase 1
CTP: cytidine triphosphate
DHAP: dihydroxyacetone phosphate
ECAR: extracellular acidification rate
FTE: Fallopian tube epithelium
FTSECs: fallopian tube secretory epithelial cells
G3P: glycerol-3-phosphate
GTP: guanosine triphosphate
HGSC: high grade serous ovarian cancer
mGPD: mitochondrial glycerol-3-phosphate dehydrogenase 2
OCR: oxygen consumption rate
UTP: uridine triphosphate
