# Supplementary material for: Metformin induces distinct bioenergetic and metabolic profiles in sensitive versus resistant high grade serous ovarian cancer and normal fallopian tube secretory epithelial cells
Source: Oncotarget. 2017 Dec 23;9(3):4044–60. doi: 10.18632/oncotarget.23661 (PMC5790520; doi:10.18632/oncotarget.23661)
Supplement: Supplementary file 1 [file oncotarget-09-4044-s001.pdf]

Metformin induces distinct bioenergetic and metabolic profiles in sensitive versus resistant high grade serous ovarian cancer and normal fallopian tube secretory epithelial cells

SUPPLEMENTARY MATERIALS

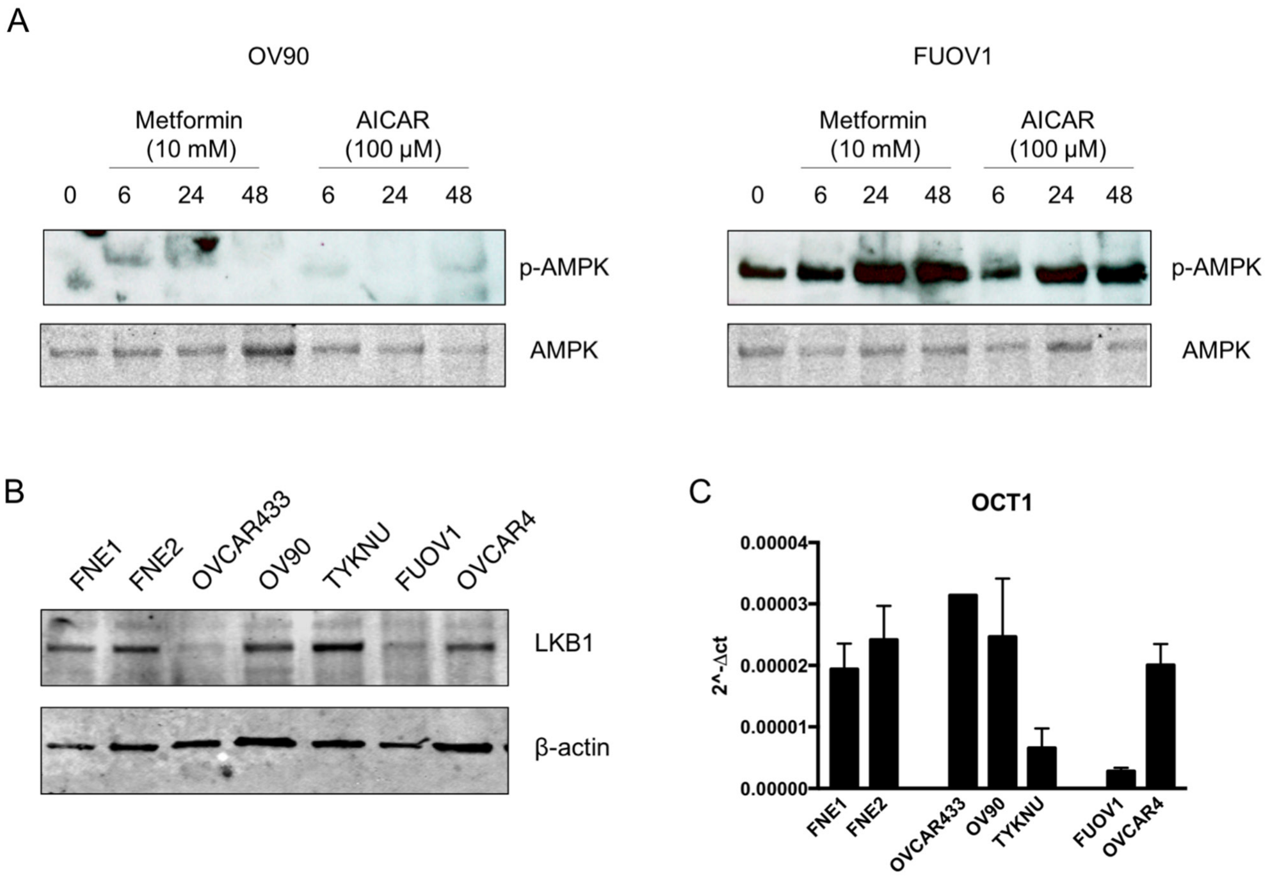

Supplementary Figure 1: Expression levels of phospho-AMPK, AMPK, LKB1, and OCT1. (A) Time course of metformin (10 mM) and AICAR (100 uM) treatment on OV90 and FUOV1. Cells were seeded and 24 hours later treated with either control, metformin, or AICAR and harvested at indicated time points. Western blot analysis for pAMPK and AMPK was performed. **(B)** Western blot analysis of LKB1 in untreated cell lines. β-actin was used as a loading control. **(C)** qRT-PCR analysis of OCT1. RPL32 transcript was used for normalization between samples.

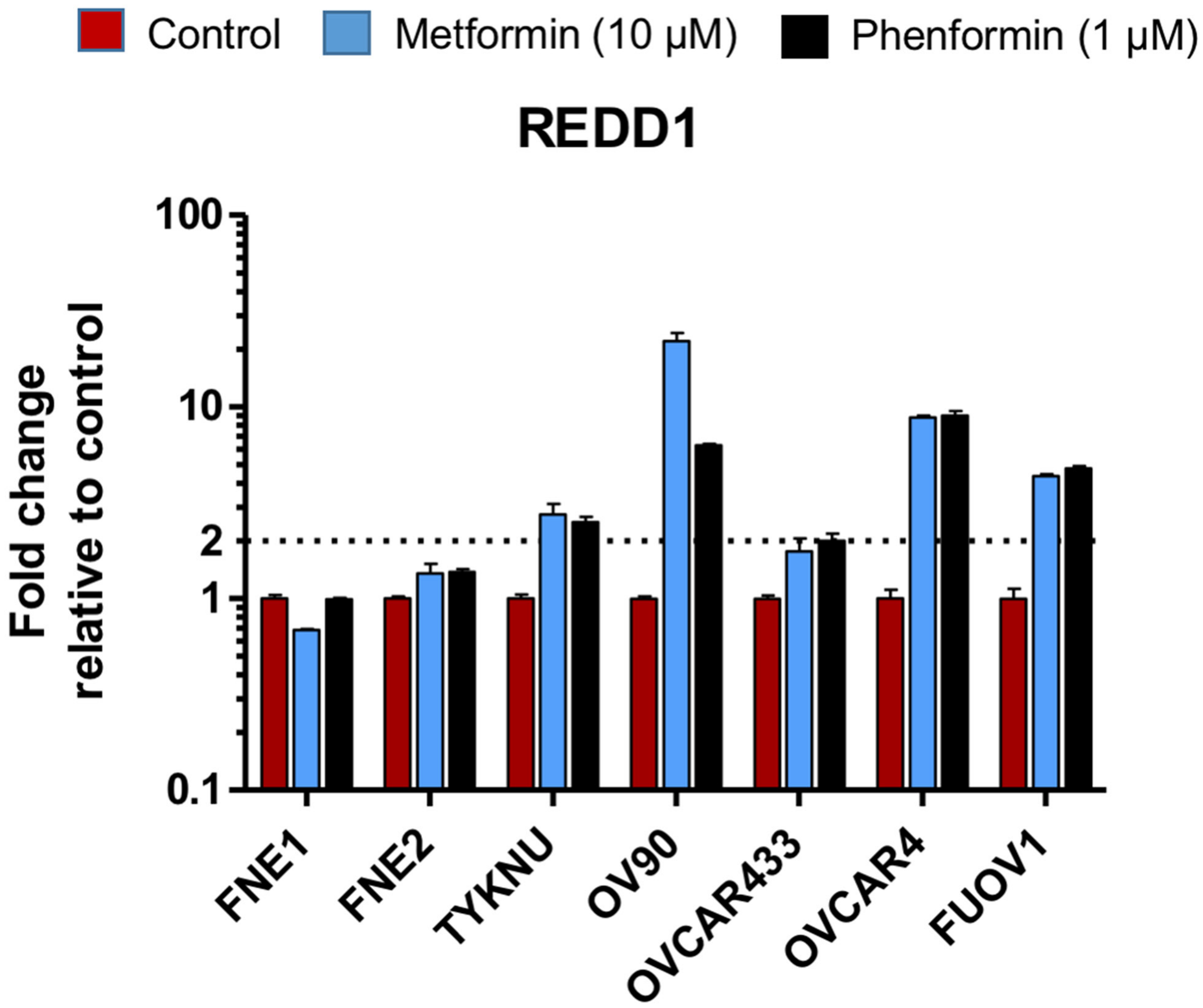

Supplementary Figure 2: The effects of biguanides on REDD1 transcription in HGSC and normal FTSEC cell lines. qRT-PCR analysis of REDD1. Fold change of each treatment shown relative to vehicle control. Dotted line indicates statistically significant upregulation (>2-fold change of a student t-test p-value < 0.01). RPL32 transcript was used for normalization between samples.

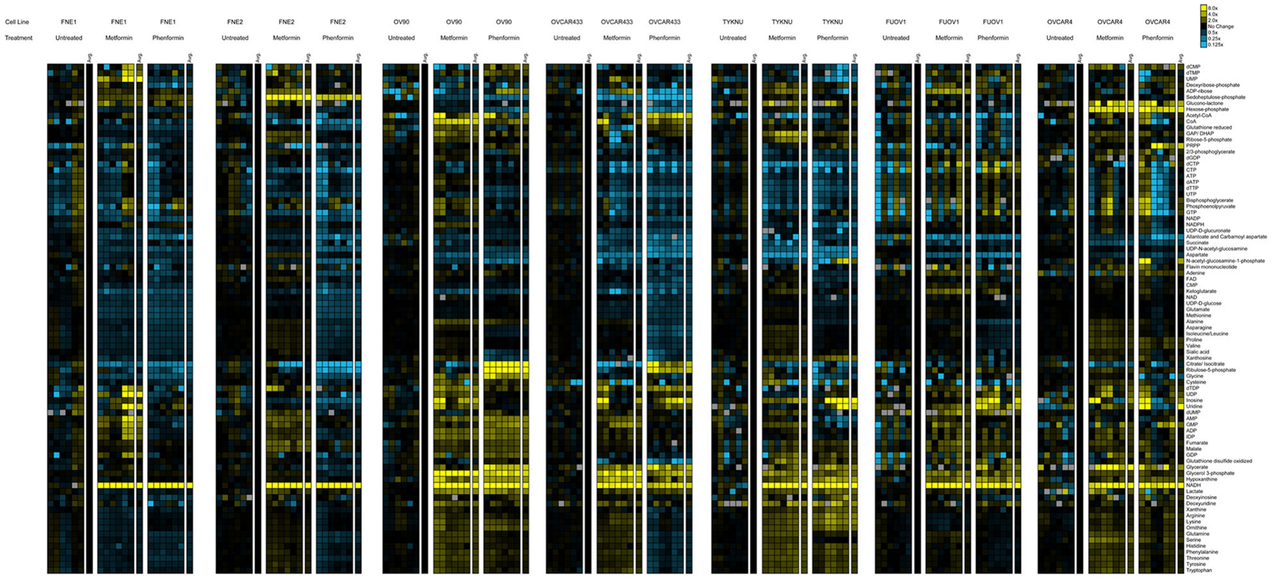

Supplementary Figure 3: Profiles of intracellular metabolites in HGSC and normal FTSEC cell lines treated with metformin (10 mM), phenformin (1 mM), or vehicle control for 24 hours. Relative levels are expressed as the log ratio of the normalized signal intensity in drug treated cells to the normalized signal intensity in the vehicle control for each cell line. Signal intensity was also normalized by cell number.

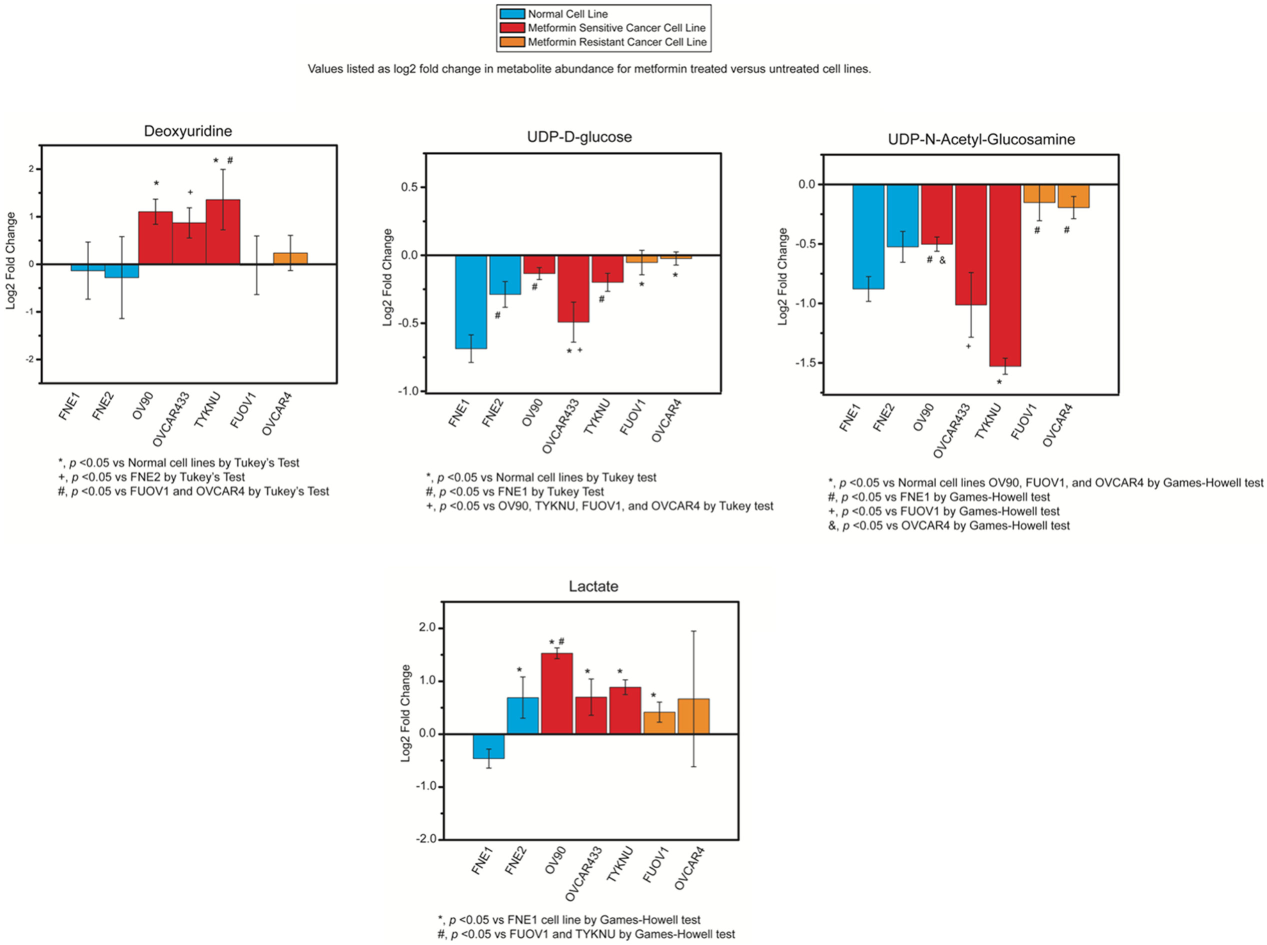

Supplementary Figure 4: Quantification of metabolite changes induced by metformin treatment. Values listed as log2 fold change in metabolite abundance for metformin treated versus control normal FTSECs (blue), metformin-sensitive (red), and metformin-resistant (orange) cells. For statistical analysis, Tukey's or Games-Howell test were used as indicated.

Supplementary Table 1: Statistical analysis of metabolic profiles shown in Figure 7 and Supplementary Figure 2 and Supplementary Figure 3. Student's *t*-test was used to determine significance. If assumption of homoscedasticity was not met, Welch's *t*-test was used instead (indicated in italics). Statistically significant differences ($p < 0.05$) are highlighted in green and underlined.

See Supplementary File 1
